# Perioperative stroke in patients undergoing spinal surgery: a retrospective cohort study

**DOI:** 10.1186/s12891-022-05591-4

**Published:** 2022-07-08

**Authors:** Xin Yan, Ying Pang, Lirong Yan, Zhigang Ma, Ming Jiang, Weiwei Wang, Jie Chen, Yangtong Han, Xiaolei Guo, Hongtao Hu

**Affiliations:** Department of Neurology, Beijing Jishuitan Hospital, Beijing, China

**Keywords:** Perioperative stroke, Spinal surgery, Incidence, Risk factor, Ischemic stroke, Hemorrhagic stroke

## Abstract

**Background:**

The incidence of perioperative stroke following spinal surgery, including ischemic and hemorrhagic stroke, has not been fully investigated in the Chinese population. Whether specific spinal or emergency/elective procedures are associated with perioperative stroke remains controversial. This study aimed to investigate the incidence of perioperative stroke, health economic burden, clinical outcomes, and associated risk factors.

**Method:**

A retrospective cohort study using an electronic hospital information system database was conducted from Jan 1, 2015, to Jan 1, 2021, in a tertiary hospital in China. Patients aged ≥18 years who had undergone spinal surgery were included in the study. We recorded patient demographics, comorbidities, and health economics data. Clinical outcomes included perioperative stroke during hospitalization and associated risk factors. The patients’ operative data, anesthetic data, and clinical manifestations were recorded.

**Result:**

A total of 17,408 patients who had undergone spinal surgery were included in this study. Twelve patients had perioperative stroke, including seven ischemic stroke (58.3%) and five hemorrhagic stroke (41.7%). The incidence of perioperative stroke was 0.07% (12/17,408). In total, 12 stroke patients underwent spinal fusion. Patients with perioperative stroke were associated with longer hospital stay (38.33 days vs. 9.78 days, *p* < 0.001) and higher hospital expenses (RMB 175,642 vs. RMB 81,114, *p* < 0.001). On discharge, 50% of perioperative patients had severe outcomes. The average onset time of perioperative stroke was 1.3 days after surgery. Stroke history (OR 146.046, 95% CI: 28.102–759.006, *p* < 0.001) and hyperlipidemia (OR 4.490, 95% CI: 1.182–17.060, *p* = 0.027) were associated with perioperative stroke.

**Conclusion:**

The incidence of perioperative stroke of spinal surgery in a tertiary hospital in China was 0.07%, with a high proportion of hemorrhagic stroke. Perioperative stroke patients experienced a heavy financial burden and severe outcomes. A previous stroke history and hyperlipidemia were associated with perioperative stroke.

## Introduction

With the increasing aging population worldwide, the number of patients with several comorbidities undergoing spine surgery has grown gradually, followed by an increase in perioperative stroke over time [1, 2]. Perioperative stroke of spinal surgery is a rare but devastating complication that is a significant source of morbidity and mortality [3]. It creates heavy financial burden on the public health care system and patients’ families.

The incidence of perioperative stroke of spinal surgery varies widely from 0.006 to 1.0% in different studies, depending on selected surgical procedures and patient populations [4–10]. Most studies have focused on ischemia rather than hemorrhagic stroke and have concentrated on specific spinal operations, such as lumbar or cervical spinal fusion. However, the true incidence of perioperative stroke, including ischemic and hemorrhagic stroke, in a broad spectrum of spinal surgeries has not been sufficiently investigated.

There are studies on the risk factors of perioperative stroke in spinal surgery, such as advanced age and diabetes [6], but whether specific spinal procedures or emergency/elective procedures are risk factors for perioperative stroke is controversial [11]. It is necessary to identify the risk factors for perioperative stroke in spinal surgery to prevent complications.

The purpose of the study was to investigate: [1] the incidence of perioperative stroke, including hemorrhagic and ischemia stroke during the hospitalization [2]; the health economic burden of perioperative stroke [3]; clinical manifestation and outcome; and [4] the associated risk factors, in a large Chinese database of spinal surgery.

## Method

### Database and patients

A retrospective cohort study using the electronic health information system (HIS) database was conducted from Jan 1, 2015 to Jan 1, 2021 in a tertiary hospital in China. Patients aged ≥18 years who had undergone spinal surgery were included in the study. This cohort study included both emergency and elective spinal surgery. Emergency surgery included spinal fracture, dislocation, and spinal cord injury requiring urgent surgery. Elective surgery included spinal fusion, disc discectomy, and percutaneous kyphoplasty, et al. The surgery site consisted of cervical, thoracic, and lumbosacral vertebrae. Patients with acute spinal trauma accompanied by severe head trauma and intraspinal tumors were excluded from the analysis. All experimental protocols were approved by the Ethics Committee of Beijing Jishuitan Hospital (No. 202004–76).

Patient demographics, risk factors, Charlson comorbidity index [12], length of hospital stay, total hospital expenses, length of intensive care unit (ICU) stay, duration of ventilator application, and blood transfusion were extracted from the database. Risk factors included hypertension, diabetes, coronary heart disease, atrial fibrillation, history of cerebral vascular diseases, hyperlipidemia, chronic obstructive pulmonary disease, and renal dysfunction.

### Outcomes

The primary outcome was perioperative stroke, which refers to stroke that occurred within 14 days after surgery during hospitalization. Perioperative stroke included acute ischemic and hemorrhagic cerebrovascular events, and was defined as rapidly developing signs of abnormal cerebral function lasting more than 24 h and confirmed by CT and/or MRI. Perioperative stroke patients were evaluated by at least two neurology attending doctors. We recorded the patient’s operative and anesthetic data, including American Society of Anesthesiologists Physical Status Classification System (ASA) score, baseline systolic blood pressure (SBP), and maximum and minimum SBP during surgery. The definition of intraoperation hypotension (IOH) was SBP < 100 mmHg and/or < 30% baseline blood pressure [13]. The time of stroke onset, clinical manifestation, and patients’ activity of daily life (modified Rankin score, mRS) were recorded. The secondary outcome was the risk factors associated with perioperative stroke.

### Statistical analysis

Statistical analyses were performed using SPSS 24.0. T-test or Kruskal–Wallis test were used to analyze continuous variables (mean ± standard deviation). Chi-square or Fisher’s exact tests were used for categorical variables (%). We performed binary logistic analysis to determine the risk factors associated with perioperative stroke, expressed as odds ratios (OR) and 95% confidence intervals (CIs). Variables that demonstrated significant differences (*p* < 0.10) in the univariate analysis were entered into logistic regression. Statistical significance was set at *P* < 0.05.

## Result

A total of 17,408 patients 8593 men, 8815 women) who had undergone spinal surgery from Jan 1, 2015 to Jan 1, 2021 at Beijing Jishuitan Hospital were included in the cohort study. The average age was 56.57 ± 14.68 years, ranging from 18 to 99 years. There were 1504 (8.7%) emergency and 15,904 (91.4%) elective surgery cases. The most common surgical level was lumbosacral (11,589; 66.6%), followed by cervical (4197; 24.1%) and thoracic (1622; 9.3%). The most common surgical procedure was spinal fusion surgery (11,804; 68.0%), followed by discectomy (2734; 15.7%), and kyphoplasty (2399; 13.8%).

Overall, perioperative stroke occurred in 12 patients (7 men, 66.50 ± 11.11 years). The incidence of postoperative stroke was 0.07% (12/17,408 patients). Seven patients (58.3%) had ischemic stroke, and five patients (41.7%) had hemorrhagic stroke (three patients had both subarachnoid and intracerebral hemorrhage; two patients had intracerebral hemorrhage). One patient died of severe complications (mortality rate of 8.3%). All 12 patients with perioperative stroke underwent spinal fusion. Among them, one patient (1/1504, 0.07%) underwent emergency surgery and 11 patients (11/15,904; 0.07%) underwent elective surgery (Table 1).

**Table 1:** Demographic, clinical, and surgical characteristics of spinal surgery patients and perioperative stroke patients (Number, (%))

	Spinal surgery	Perioperative stroke	*P* value
Number	17,396	12	
Gender			1.000
Male	8586 (49.9%)	7 (58.3%)	
Female	8810 (50.1%)	5 (41.7%)	
Age (years), mean [SD]	56.56 [14.77]	66.50 [11.11]	0.020
18–40	2660 (15.3%)	0	
41–60	6876 (39.5%)	2 (16.7%)	
61–80	7320 (42.1%)	10 (83.3%)	
≥ 81	540 (3.1%)	0	
Level of spinal surgery			0.168
Cervical	4195 (24.1%)	2 (16.7%)	
Thoracic	1619 (9.3%)	3 (25.0%)	
Lumbosacral	11,582 (66.6%)	7 (58.3%)	
Surgery procedure			0.223
Spinal fusion	11,804 (68.0%)	12 (100%)	
Disc discectomy	2734 (15.7%)	0	
Kyphoplasty	2399 (13.8%)	0	
Other	459 (2.6%)	0	
Surgery Type			0.970
Emergency	1503 (8.6%)	1 (8.3%)	
Elective	15,893 (91.4%)	11(91.7%)	
Blood Transfusion			
Red Blood Cell	534 (3.1%)	4 (33.3%)	< 0.001
Volume U, mean [SD]	3.88 [3.24]	4 [1.63]	0.939
Platelet	13 (0.1%)	0	1.000
Volume U, mean [SD]	1.77 [1.23]	0	/
Plasma	299 (1.7%)	4 (33.3%)	< 0.001
Volume U, mean [SD]	5.89 [4.73]	10.50 [8.54]	0.056
ICU stay	1658 (9.24%)	7 (58.33%)	< 0.001
Duration (hour), mean [SD]	51.34 [587.97]	265 [244.47]	0.337
Ventilator Usage	1299 (7.47%)	4 (33.3%)	0.009
Duration (hour), mean [SD]	29.69 [660.79]	109.50[159.12]	0.809
Charlson comorbidity index, mean [SD]	0.26 [0.73]	1.58 [1.56]	< 0.001
Length of hospital stay (days), mean [SD]	9.78 [9.04]	38.33 [55.84]	< 0.001
Total Hospital Cost (RMB), mean [SD]	81,113.88[52,969.84]	175,649.18 [90,990.88]	< 0.001

t test and X^2^ test were used to analyze

Perioperative stroke patients were significantly older (66.5 years vs. 56.6 years, *p* < 0.05) and had a greater comorbidity burden (Charlson comorbidity index:1.58 vs. 0.26, *p* < 0.001) than unaffected patients, with longer length of hospital stay (38.33 days vs. 9.78 days, p < 0.001) and higher hospital cost (RMB 175,649 vs. RMB 81,114, p < 0.001). Patients who developed perioperative stroke had a higher risk of intensive care unit need (58.33% vs. 9.24%, p < 0.001), ventilator use (33.33% vs. 7.47%, *p* < 0.01), plasma transfusion (33.3% vs.1.7%, p < 0.001), and red blood cell transfusion (33.3% vs. 3.1%, p < 0.001) than unaffected patients (Table 1). The median surgical procedure time was 165 min, and four patients (three ischemic stroke and one hemorrhagic stroke) had intraoperative hypotension (Table 4).

The average time of stroke onset was 1.3 days after spinal procedures, ranging from 0 to 5 days. Hemorrhagic stroke occurred earlier (0.4 days, 0–1 day) than ischemic stroke (2.2 days, 0–5 days) (*p* < 0.05). None of the 12 perioperative patients had received antiplatelet or anticoagulation therapy within 14 days. The most common clinical symptoms were consciousness disorder (8 patients, 66.7%), dysarthria (5 patients, 60%), and paralysis (5 patients, 60%). The NIHSS score was 14.60 ± 11.10 (2–34) on day 0, and 9.90 ± 11.59 (0–39) on the 14th day, without significant improvement (*p* = 0.376). On discharge, six patients (50%) had severe outcomes, with modified Rankin score > 3 (Table 4).

The chi-square and univariate analyses identified that the significant risk factors for perioperative stroke were age ≥ 65 years (66.7% vs. 32.1%, *p* = 0.010), hypertension (66.7% vs. 25.4%, *p* < 0.01), hyperlipidemia (33.3% vs. 5.0%, p < 0.01), stroke history (83.3% vs. 1.9%, *p* < 0.001), Charlson comorbidity index ≥3 (83.3% vs. 0.6%, p < 0.001), spinal fusion procedure (100% vs. 67.8%, *p* = 0.017), red blood transfusion (33.3% vs. 3.1%, p < 0.001), and plasma transfusion (33.3% vs. 1.7%, p < 0.001) (Table 2). Binary logistic regression demonstrated that stroke history (OR 146.046, 95% CI: 28.102–759.006, p < 0.001) and hyperlipidemia (OR 4.490, 95% CI: 1.182–17.060, *p* = 0.027) were independent predictors for the development of perioperative stroke (Table 3).

**Table 2:** Risk factors of spinal surgery patients and perioperative stroke patients (Number (%))

Risk factors	Spinal surgery	Perioperative stroke	P value
Age			0.010
< 65 years	11,804 (67.9%)	4 (33.3%)	
≥ 65 years	5592 (32.1%)	8 (66.7%)	
Spinal fusion	11,792 (67.8%)	12 (100%)	0.017
Other procedure	5604 (32.2%)	0	
Cervical level	4197 (24.1%)	2 (16.7%)	0.546
Other level	13,199 (75.9%)	10 (83.3%)	
Hypertension	4425 (25.4%)	8 (66.7%)	0.003
Hyperlipidemia	875 (5.0%)	4 (33.3%)	0.002
Coronary heart disease	849 (4.9%)	1 (8.3%)	0.452
Atrial fibrillation	86 (0.5%)	0	1.000
Acute heart infarction	192 (1.1%)	0	1.000
Chronic heart disease	22 (0.1%)	0	1.000
Diabetes	1,890 (10.9%)	2 (0.1%)	0.381
Stroke history	336 (1.9%)	10 (83.3%)	< 0.001
Chronic lung disease	150 (0.9%)	0	1.000
Renal disease	84 (0.5%)	1(8.3%)	0.057
Charlson comorbidity index			< 0.001
0–2	17,292 (99.4%)	2 (16.7%)	
≥ 3	104 (0.6%)	10 (83.3%)	

X^2^ test was used to analyze

**Table 3:** Binary logistical regression analysis of risk factors for perioperative stroke

	Odds ratio (OR)	95% confidence interval	P value
Age ≥ 65 years	2.138	0.586–7.796	0.250
Hypertension	0.942	0.245–3.622	0.931
Hyperlipidemia	4.490	1.182–17.060	0.027
Stroke history	146.046	28.102–759.006	< 0.001
Renal disease	1.842	0.452–7.502	0.394
Charlson comorbidity index≥3	0.166	0.023–1.213	0.077
Spinal fusion procedure	0.000	< 0.001	0.974

**Table 4:** Clinical status of perioperative stroke patients of spinal surgery

No	S E X	A G E	Comorbidity	Diagnosis	Procedure	Dura- tion (min)	ASA	Blood Transfusion	Base SBP (mm Hg)	Max SBP (mm Hg)	Min SBP (mmHg)	IOH	Clinical Manifestation	Imaging	mRS	LOS (days)
1	F	61	HT,HLP	Lumbar degenerative disc disease (L5/S1)	Posterior lumbar Spinal fusion	120	II	No	130	130	110	no	dizziness, dysarthria, dysphagia, hemiparalysis	Ischemia stroke, large artery stenosis	3	36
2	M	68	HT, CVD	lumbar spinal stenosis (L2/3, L4/5)	Posterior lumbar Spinal fusion	180	II	No	160	160	100	yes	somnolence, dysarthria, ataxia, facial palasy, hemisensory loss	Ischemia stroke, large artery stenosis	4	29
3	F	69	HT, DM	lumbar spinal stenosis (L3/4, L5/S1)	Transforaminal lumbar interbody fusion	180	II	No	170	170	105	yes	apathy, hemiparalysis, dysphasia, facial palsy	Ischemia stroke, large artery stenosis	4	25
4	M	48	HT	Cervical degenerative disc disease (C3/4)	Anterior cervical spianl fusion	90	II	No	140	140	110	no	apathy, hemiparalysis, gaze	Ischemia stroke, large artery stenosis	2	20
5	M	44	Smoking	Thoracic spine fracture and dislocation (T11, T12)	Thoracic spinal fusion	270	IV	No	120	120	105	no	dysarthria	Ischemia stroke	5	15
6	M	78	CVD	Lumbar spinal stenosis (L4/5)	Posterior lumbar spinal fusion	120	II	Yes	140	140	100	no	confusion, hemiparalysis	Hemorrhage stroke	5	203
7	F	80	HT	Ossification of thoracic ligamentum flavum (T1–3)	Posterior thoracic spinal fusion	180	IV	Yes	130	130	70	yes	somnolence, dysarthria, facial palasy	SAH, hemorrhage stroke	1	13
8	F	68	HT	Cervical degenerative disc disease (C3–6)	Posterior cervical spinal fusion	120	II	No	130	130	110	no	somnolence, vomitting, nystagmus, dysarthria, ataxia	SAH, hemorrhage stroke	4	38
9	F	74	HT, HLP	Ossification of thoracic ligamentum flavum (T10/11)	Posterior thoracic spinal fusion	210	III	Yes	145	145	90	yes	coma, dizziness, vomiting, dysphasia, gaze	Hemorrhage stroke	6	23
10	M	68	HT, DM	Lumbar spondylolisthesis L4; degenerative disc disease (L5/S1)	Posterior cervical spinal fusion	185	II	Yes	175	175	125	no	Coma, neck rigidity	SAH, hemorrhage stroke	3	53
11	F	64	HLP	Lumbar spondylolisthesis; spinal stenosis(L4/5, L5/S1)	Transforaminal lumbar interbody fusion	150	II	No	110	110	100	no	hemiparalysis	Ischemia stroke	2	23
12	M	76	CVD, HT, HLP CHD	lumbar spinal stenosis (L4/5)	Transforaminal lumbar interbody fusion	90	III	No	165	165	120	no	dizziness, vomiting, dysarthria, hemiparalysis	Ischemia stroke	1	12

*HT* hypertension, *HLP* hyperlipidemia, *DM* diabetes mellitus, *CVD* previous cerebral vascular disease, *CHD* coronary heart disease, *ASA* American Society of Anesthesiologists physical status classification system, *SBP* systolic blood pressure, *MAX SBP* Maximum systolic blood pressure during operation, *MIN SBP* Minimum systolic blood pressure during operation, *IOH* intraoperative hypotension, < 100 mmHg and/or < 30% baseline blood pressure, *mRS* modified Rankin Scale, *LOS* length of hospital stay

## Discussion

The incidence of perioperative stroke of spinal surgery was 0.07% in 17,408 patients at a tertiary hospital in China. To our knowledge, this study is the first to investigate the incidence of stroke following all spinal procedures, both elective and emergency. The incidence of perioperative stroke in spinal surgery varies widely, from 0.01 to 1.0%, depending on the surgical technique and patient population [1]. An American study found that the incidence of postoperative stroke was 0.05% in 13,660 patients after spinal surgery [4]. In a Swedish study of 5029 patients in a large tertiary referral center, the incidence of ischemic stroke was 0.15% after elective spine surgery [7]. A Japanese study of 167,106 elective spinal surgery patients found that the incidence of perioperative stroke was 0.22% [9]. In some spinal surgery, the incidence is much higher. An American retrospective study of 43,063 patients with elective posterior lumbar fusion demonstrated an incidence of 0.29% [6]. In a Japanese emergency cervical spinal injury study of 11,005 patients, the incidence of ischemic stroke was as high as 1.0% [10].

Perioperative stroke is a rare complication but represents a tremendous burden. The stroke patients had almost four times the length of hospital stay (38.33 days vs. 9.78 days) and twice the total hospital costs (RMB 175,649 vs. RMB 81,113) compared with non-perioperative stroke patients. These findings are consistent with the published literature. Alejandro et al. found that the length of hospital stay (8.9 days vs. 3.9 days) and total hospital cost ($41,454 vs. $25,885) of postoperative patients were twice those of unaffected patients in a 10-year spinal fusion study among 264,891 patients in the USA [14]. Futhermore, the personal impact of perioperative stroke has been devastating. The stroke patients had six times higher incidence of ICU stay (58.33% vs. 9.24%) and four times higher ventilator usage (33.33% vs. 7.47%). Our study demonstrated that half of the patients had severe disabilities upon discharge, which caused a heavy care and financial burden to the patient’s family and health system. Similar results were demonstrated that 43% perioperative stroke patients suffered from neurological deficits during hospital stay [7].

The mean time of cerebral event onset was 1.3 days (0–5 days), similar to our previous study. Most perioperative strokes appear within the first 3 days, especially within the first 24 hours [15–17]. The signs and symptoms of perioperative stroke could be confused with delayed anesthesia recovery, pain, and complications of spinal surgery, such as nerve injury or spinal hematoma, thus neglecting possible cerebral vascular events [7]. Therefore, high-risk patients should be closely monitored during the first 3 days after surgery. Emergency CT or MRI should be completed immediately if perioperative stroke is suspected.

Ischemic stroke is more common than hemorrhagic stroke in most perioperative cases [18]. It was reported that the incidence of hemorrhagic stroke was up to 5% for different types of surgery [3]. However, in our study, hemorrhagic stroke was as high as 41.7% (5/12). A similar result was demonstrated in another Japanese spine surgery study of 167,106 patients; the hemorrhagic stroke rate was relatively high (14.3%) [9]. Among the five patients with hemorrhage in our study, two had definite dura mater tearing and one had possible dura mater tearing. Intracranial hemorrhage after spinal surgery has been reported following the loss of cerebrospinal fluid due to dural tear [19–21]. Instrumented fusion and pedicle screwing could increase the risk of intraoperative dural lesions, leading to postoperative CSF leakage and intracranial hypotension, which could induce a downward sag of the cerebellum and result in rupture of blood vessels [22, 23]. Thus, it is necessary to avoid tearing of the dura mater during the operation. If the dura mater is damaged, it should be repaired immediately, and attention should be paid to the amount of the cerebral spinal fluid drainage.

A third of perioperative stroke patients received blood transfusions for hemorrhagic complications of spinal surgery, due to exposure of the bone and stripping of muscles. Blood transfusion is related to prolonged surgery duration and a large amount of blood loss, and is an independent predictor of mortality and morbidity [24, 25].

The published literature has demonstrated that age, renal disease, atrial fibrillation, history of stroke, cardiac valvular disease, etc., are risk factors for perioperative stroke, but has offered little insight regarding the risk factors unique to spinal surgery [2, 6, 26]. Our study found that a previous stroke history was a significant risk factor (OR = 146.046) for perioperative stroke in both elective and emergency spinal surgery. A similar result was demonstrated in an elective posterior lumbar fusion study of 43,063 patients in the USA [6]. There are two possible reasons for this. First, discontinuation of antiplatelet or anticoagulant drugs and the hypercoagulable state after the operation can induce stroke [2]. Second, patients with a history of stroke have impaired cerebrovascular autoregulation and decreased embolism clearance [27]. Cerebral vascular events can occur when blood pressure decreases during surgery. Routine vascular evaluation should be performed before surgery to avoid perioperative stroke, including carotid ultrasound and transcranial Doppler ultrasound (TCD) in high-risk patients [3, 17, 28].

Hyperlipidemia was another risk factor for perioperative stroke (OR 4.490). A single-center study of 5029 patients who underwent elective spinal surgery demonstrated similar results [7]. Statins should be continued in patients currently receiving statins and undergoing surgery [3, 16]. Recognizing high-risk patients and providing a multidisciplinary plan are essential to reduce the risk of postoperative stroke [16]. Although all 12 patients with perioperative stroke underwent spinal fusion, we did not find an association between the procedure and perioperative stroke. In contrast, another elective spinal surgery study of 167,106 patients reported that spinal tumor resection and cervical spinal surgery were risk factors for perioperative stroke [9]. Further studies are needed to investigate the correlation between spinal procedures and perioperative stroke.

Our study had some limitations. First, we only calculated the patients with perioperative stroke after surgery during hospitalization within 14 days. Stroke that occurred after discharge could have been missed in this study. However, most strokes occurred early following surgery, and the median postoperative day was 2–9 days [29]. In our study, the average length of hospital stay was almost 10 days, which covers the majority of strokes. Second, our study was a single-center study in a large academic hospital; thus, a selection bias could exist. A multicenter study including non-academic hospitals should be conducted in the future. Third, patients’ signs and symptoms of stroke could be confused by sedative use, pain, and spinal surgery complications; thus, silent or mild ischemic stroke may have been overlooked. The actual incidence of perioperative stroke could be high [30]. Fourth, the number of identified perioperative stroke was small, which was the main limitation of this study. As the entire cohort of spinal surgery patients was very large, it reflected the rarity of the complication. However, because of the small sample size, it is difficult to draw definite conclusions about the cause and effect. Lastly, we did not calculate all intra-operative data, such as surgery duration, intraoperative blood pressure, and blood loss volume, which should be further investigated in future studies.

## Conclusions

The incidence of perioperative stroke of spinal surgery in a Chinese population was 0.07%. Perioperative stroke patients had a lengthy hospital stay and high hospital expenses, with severe discharge outcomes. Hemorrhagic stroke was present in a high proportion of patients. Previous stroke history and hyperlipidemia were risk factors for perioperative stroke. Our study provides useful information for patient counseling and perioperative stroke prevention.

## Data Availability

The data that support the findings of this study are available from health information system, but restrictions apply to the availability of these data, which were used under license for the current study, and so are not publicly available. Data are however available from the corresponding author (Xin Yan) upon reasonable request and with permission of health information system.
